# Pulmonary hypertension without heart failure causes cardiorenal syndrome in a porcine model

**DOI:** 10.1038/s41598-023-36124-1

**Published:** 2023-06-05

**Authors:** Arthur Orieux, Chloé Samson, Laurence Pieroni, Sarah Drouin, Simon Dang Van, Tiffany Migeon, Perrine Frere, Dorothée Brunet, David Buob, Juliette Hadchouel, Julien Guihaire, Olaf Mercier, Pierre Galichon

**Affiliations:** 1 INSERM UMR_S1155 Bâtiment Recherche, CoRaKiD, Hôpital Tenon, 4 Rue de La Chine, 75020 Paris, France; 2 Sorbonne Université, Paris, France; 3 AP-HP Hôpital Tenon - Service de Biochimie, Paris, France; 4 Service Médico Chirurgical de Transplantation Rénale, AP-HP Hôpital Pitié Salpêtrière, Paris, France; 5 INSERM UMR_S999, Hôpital Marie Lannelongue - Groupe Hospitalier Paris Saint Joseph, Le Plessis Robinson, France; 6 Université Paris-Saclay, Le Kremlin-Bicêtre, France; 7 Service de Chirurgie Thoracique et Transplantation Cardio-Thoracique, Hôpital Marie Lannelongue - Groupe Hospitalier Paris Saint Joseph, Le Plessis Robinson, France; 8 AP-HP Hôpital Tenon - Service d’Anatomie Pathologique, Paris, France

**Keywords:** Physiology, Cardiology, Medical research, Nephrology

## Abstract

Cardiorenal syndromes type 1 and 2 are complex disorders in which cardiac dysfunction leads to kidney dysfunction. However, the mechanisms remain incompletely explained, during pulmonary hypertension in particular. The objective of this study is to develop an original preclinical model of cardiorenal syndrome secondary to a pulmonary hypertension in piglets. Twelve 2-month-old Large White piglets were randomized in two groups: (1) induction of pulmonary hypertension by ligation of the left pulmonary artery and iterative embolizations of the right lower pulmonary artery, or (2) Sham interventions. We evaluated the cardiac function using right heart catheterization, echocardiography and measurement of biochemistry markers). Kidney was characterized using laboratory blood and urine tests, histological evaluation, immunostainings for renal damage and repair, and a longitudinal weekly assessment of the glomerular filtration rate using creatinine-based estimation and intravenous injection of an exogenous tracer on one piglet. At the end of the protocol (6 weeks), the mean pulmonary artery pressure (32 ± 10 vs. 13 ± 2 mmHg; *p *= 0.001), pulmonary vascular resistance (9.3 ± 4.7 vs. 2.5 ± 0.4 WU; *p *= 0.004) and central venous pressure were significantly higher in the pulmonary hypertension group while the cardiac index was not different. Piglets with pulmonary hypertension had higher troponin I. We found significant tubular damage and an increase in albuminuria in the pulmonary hypertension group and negative correlation between pulmonary hypertension and renal function. We report here the first porcine model of cardiorenal syndrome secondary to pulmonary hypertension.

Cardiorenal syndromes (CRS) can be generally defined as a pathophysiologic disorder of the heart and kidneys whereby an acute or chronic dysfunction of one organ induces the acute or chronic dysfunction of the other^1^. CRS are responsible for significant morbidity and mortality^2^. They have been categorized into five clinical subtypes based on the primary organ affected and its acute or chronic character for descriptive purposes. However, the pathophysiological pathways involved are common and suggest an unified pathogenesis^3^. Since the recognition of CRS as an entity, the majority of the studies that described its pathophysiology assessed patients with left heart failure (LHF) and reduced ejection fraction. However, clinical and preclinical data evaluating renal dysfunction secondary to pulmonary hypertension (PH) are scarce, making it an unrecognized cause of CRS^4^. In addition, there is a lack of data regarding chronic PH and right ventricular chronic pressure overload as a cause of CRS. PH is a syndrome characterized by a pulmonary vascular resistance higher than 3 Wood Units (WU), associated with a mean pulmonary artery pressure (mPAP) greater than 20 mmHg (pre-capillary PH forms)^5^.

The cause of the kidney injury observed in patients with PH due to a thromboembolic disease (group 4 PH) is complex because left ventricular ejection fraction (LVEF) is commonly preserved in chronic thromboembolic pulmonary hypertension, except in case of intrinsic left ventricular cardiomyopathy (ischemic, valvular, rhythmic). Like LHF, the venous congestion [elevated right atrial pressures RAP)] probably plays an essential role in the renal dysfunction in patients with PH and right ventricular chronic pressure overload^6,7^. Moreover, these patients may have neurohormonal activation, of the renin–angiotensin–aldosterone system (RAAS) for example, with impaired natriuresis and reduced renal blood flow (RBF)^8^.

There are few animal models of CRS and they are mainly developed to assess the LHF consequences on the kidney. Unlike mouse or rat models, large animal models of heart failure have some advantages in terms of clinical translation due to many similarities with humans and allow more precise hemodynamic monitoring with clinical-grade equipment. Importantly, many models of pulmonary hypertension like hypoxia, VEGF inhibition and toxicants like monocrotaline cause direct kidney injury, which precludes evaluating the consequences of pulmonary hypertension on the kidney*.* Thus, there is a need to develop new surgical models on large animals to improve the characterization of the cardiorenal interaction and to approve new clinical therapeutics or medical devices^9^.

The objective of this study was to develop a piglet preclinical model of CRS secondary to PH due to a thromboembolic disease and characterize its consequences.

## Results

### Induction of pulmonary hypertension in piglets and right ventricular remodeling

As expected, the mPAP, PVR and central venous pressure increased after 6 weeks in piglets from the PH group compared to the Sham group (Table 1). However, the cardiac index (CI) was comparable between groups. We found a right ventricle systolic dysfunction in piglets with PH as evidenced by a significant decrease in tricuspid annular plane systolic excursion (TAPSE) (6 [7–14] vs. 12 [13–16] mmHg; *p *= 0.03), whereas the systolic function of the left ventricle was not different between the two groups. Compared with piglets from the Sham group, cardiac troponin I was higher at 6 weeks in the PH group (303 [53–3054] vs 33 [17–47] ng/mL); *p *= 0.03).

**Table 1:** Pulmonary hypertension induction in piglets and cardiac consequences.

	PH (*n* = 6)	SHAM (*n* = 6)	*p-*value
Clinical parameters			
Weight at D0 (kg)	22.9 ± 2	23.7 ± 2.7	0.59
Weight at sacrifice (kg)	31.3 ± 5	33.2 ± 2.9	0.43
Weight gain between D0 and sacrifice at W6 (kg)	8.4 ± 3.1	9.5 ± 3.1	0.54
Body surface area (BSA) at sacrifice (cm^2^)	7004 ± 751	7299 ± 409	0.42
Hemodynamic parameters			
Heart rate (bpm)	107 [96–130]	106 [80–140]	0.82
Mean pulmonary artery pressure (mPAP) (mmHg)	32 ± 10	13 ± 2	0.001
Central venous pressure (CVP) (mmHg)	10 ± 4	6 ± 2	0.04
Total pulmonary resistance (TPR) (WU)	13.8 ± 7	4.1 ± 0.6	0.006
Pulmonary vascular resistance (PVR) (WU)	9.3 ± 4.7	2.5 ± 0.4	0.004
Cardiac index (CI) (L/min/m^2^)	2.6 ± 0.8	3.3 ± 0.4	0.09
Echocardiography parameters			
LVEF (%)	69.6 ± 5.5	67.9 ± 3	0.23
Tricuspid S’ peak systolic velocity (cm/s)	5 [4–8]	9 [8–10]	0.09
TAPSE (cm)	7 [6–14]	13 [12–16]	0.03
Biologic parameters			
AST (UI/L)	58 ± 18	45 ± 13	0.18
CK (UI/L)	907 ± 291	723 ± 191	0.23
LDH (UI/L)	695 ± 237	540 ± 118	0.18
Troponin I (ng/mL)	303 [53–3054]	33 [17–47]	0.03

Evaluation of clinical, hemodynamic, echocardiographic and biological cardiac parameters at the time of sacrifice.

*AST: aspartate aminotransferase; bpm: beats per minute; BSA: body surface area; CK: creatine kinase; LDH: lactate dehydrogenase; LVEF: left ventricular ejection fraction; mPAP: mean pulmonary artery pressure; PH: pulmonary hypertension; PVR: pulmonary vascular resistance; TAPSE: tricuspid annular plane systolic excursion; TPR: total pulmonary resistance; VCP: venous central pressure.*

No statistically significant difference is observed for the hemodynamic data (mPAP, TPR, PVR, CI) measured during the baseline RHC. Left ventricular systolic function (LVEF) and right ventricular systolic function (assessed by TAPSE) was not different between the PH and SHAM groups at baseline TTE (Supplementary material, Table S1).

### Pulmonary hypertension induces a renal dysfunction in piglets

At the end of the protocol, blood urea and SCr was not significantly different between the two groups (Table 2). However, piglets with PH had a reduced urea excretion fraction, a marker of renal hypoperfusion, and a modestly increased albuminuria (Table 2), compared to the Sham group.

**Table 2:** Renal biological data.

	PH (*n* = 6)	SHAM (*n* = 6)	*p*-value
Blood analysis			
Sodium (mmol/L)	139.1 ± 2.6	138.6 ± 2.1	0.70
Potassium (mmol/L)	3.5 ± 0.2	3.8 ± 0.2	0.11
Chloride (mmol/L)	99.5 ± 3.5	99.8 ± 1.3	0.94
Bicarbonate (mmol/L)	28.9 ± 3.4	28.9 ± 1.7	0.98
Calcium (mmol/L)	2.28 ± 0.1	2.37 ± 0.12	0.17
Phosphorus (mmol/L)	2.74 ± 0.29	2.51 ± 0.11	0.10
Plasma urea (mmol/L)	3.3 ± 1.9	3.2 ± 0.8	0.88
Serum creatinine (SCr) (µmol/L)	99 ± 8.3	97.8 ± 5	0.82
SCr to weight ratio (µmol/L/kg)	3.26 ± 0.78	2.94 ± 0.23	0.36
SCr to BSA ratio (µmol/L/cm^2^)	1.43 ± 0.25	1.34 ± 0.11	0.40
eGFR (mL/min/kg)	5.16 ± 1.02	5.52 ± 0.34	0.43
Total protein (g/L)	45.3 ± 3.3	45.1 ± 3	0.89
Urine analysis			
Natriuresis (mmol/L)	65 ± 31	62 ± 30	0.90
Kaliuresis (mmol/L)	78 ± 33	90 ± 28	0.52
Chloruresis (mmol/L)	44 ± 39	64 ± 29	0.32
Urine creatinine (mmol/L)	14.6 ± 5.3	11 ± 3.5	0.20
Urine urea (mmol/L)	100 ± 91	97 ± 43	0.94
Fractional excretion of urea (FE_Urea)_ (%)	18.2 ± 6.2	26.8 ± 6.2	0.04
Fractional excretion of sodium (Fe_Na_) (%)	3.4 ± 1.6	4.7 ± 3.1	0.40
Albuminuria (mg/L)	4.4 ± 0.6	2.9 ± 0.4	< 0.001
Urine albumin to creatinine ratio (UACR) (mg/mmol)	0.45 ± 0.22	0.27 ± 0.08	0.09

Characterization of renal injury by blood and urine sample, at the time of sacrifice.

*BSA: body surface area; eGFR: estimated glomerular filtration rate; FeNa: fractional excretion of sodium; FeUrea: fractional excretion of urea; PH: pulmonary hypertension; SCr: serum creatinine; UACR: urine albumin to creatinine ratio.*

**Figure 1:**
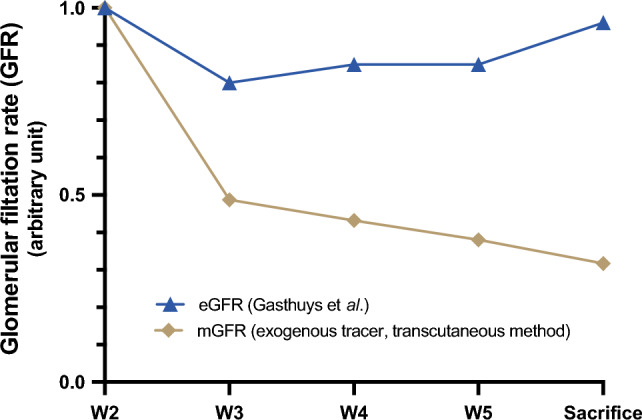
Evolution of the estimated glomerular filtration rate (eGFR) and measured (relmapirazin, mGFR), as a function of time. An evaluation of the GFR is carried out with an exogenous tracer (relmapirazin, mGFR) or by estimation from the weight and the plasma creatinine according to the formula of Gasthuys et al. (eGFR). The mGFR or the eGFR at W2 is used as a reference. *eGFR: estimated glomerular filtration rate; mGFR: measured glomerular filtration rate.*

The eGFR was not significantly different between the two groups. In addition, we measured the GFR in one piglet of the PH group every week for a month before euthanasia using the transdermal detection of a fluorescent tracker freely and fully filtered by the kidney. Unlike the eGFR (estimated from SCr and weight), the measured GFR decreased by 50% after one week, whereas eGFR decreased by only 20% (Fig. 1). Then measured GFR progressively decreased to 30% of the initial value at the time of sacrifice (on the sixth week) whereas eGFR remained stable.

### Pulmonary hypertension induces kidney epithelial injury in piglets

**Figure 2:**
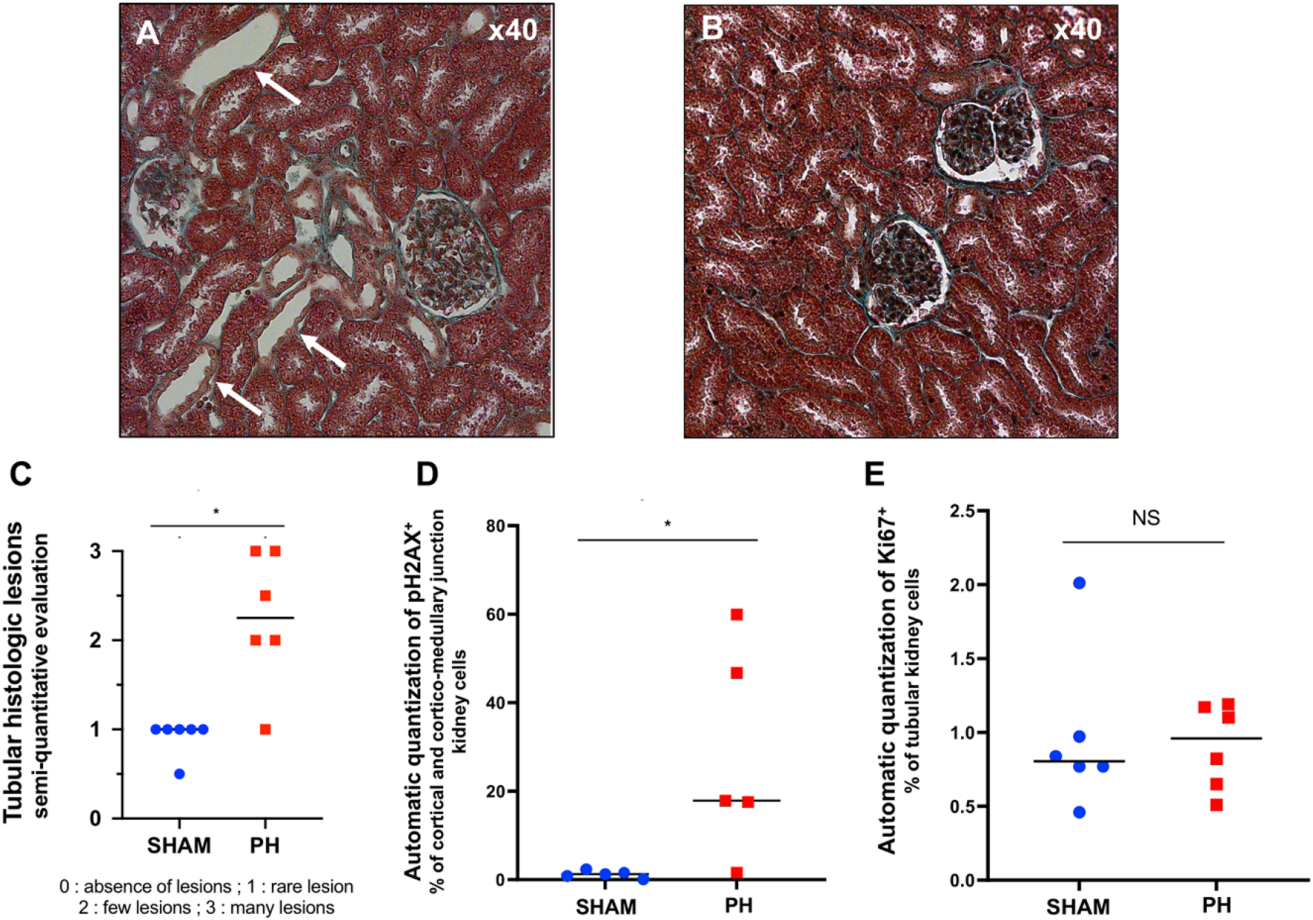
Evaluation and quantification of tubular lesions (Masson's trichrome), automatic quantization of pH2AX and Ki67 in kidney cells. (**A**): Histological section of kidney in a PH piglet, Masson's Trichrome staining (white arrow: luminal dilation and epithelial thinning). (**B**) Histological section of kidney in a SHAM piglet, Masson's Trichrome staining. (**C**) Semi-quantitative evaluation (0 to 3) of tubular lesions by a pathologist, blinded. (**D**) Automatic quantization of pH2AX in cortex and cortico-medullary junction in kidney cells (expressed in percentage of positive cells). (**E**) Automatic quantization of Ki67 in tubular kidney cells (expressed in percentage of positive cells). *PH: pulmonary hypertension.*

The analysis of the renal structure evaluated by a pathologist (Fig. 2A,B) reported a semi-quantitative score of the tubular injury lesions of acute tubular necrosis (ATN) significantly greater in the PH group than in the Sham group (Fig. 2C).

Expression of pH2AX was significantly higher in the cortex and cortico-medullary junction in kidney cells of the PH group compared to the Sham group (automatic quantization with QuPath, Fig. 2D and Figure S1). The tubular expression of Ki67 was not different between the two groups (Fig. 2E).

### Correlation between the degree of pulmonary hypertension and renal dysfunction

**Figure 3:**
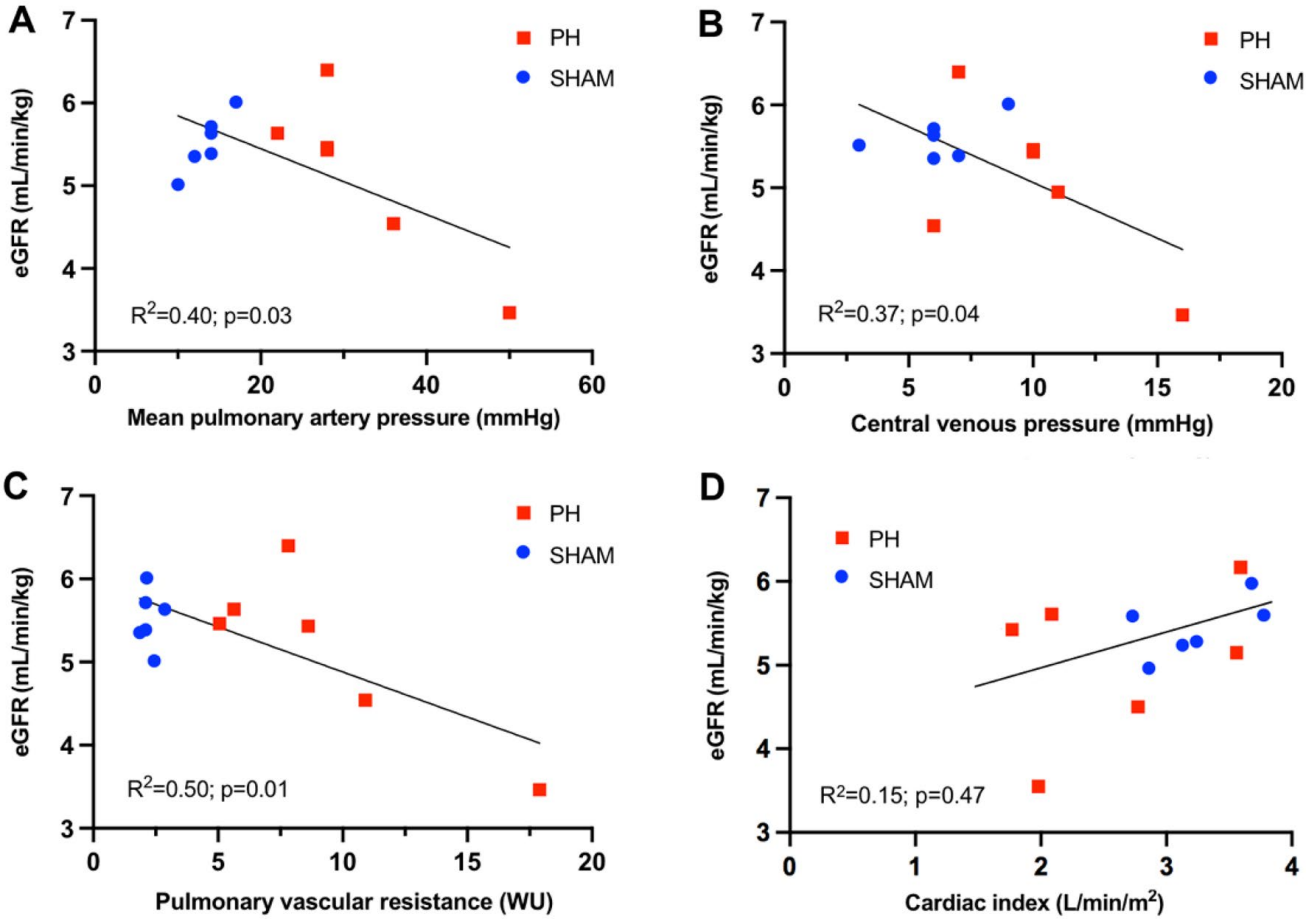
Correlation between hemodynamics parameters of PH and renal function. Mean pulmonary arterial pressure (mPAP). (**A**) central venous pressure (CVP) (**B**), and pulmonary vascular resistance (PVR) (**C**) at sacrifice were correlated with renal dysfunction (estimated GFR), unlike cardiac index (CI) (**D**). *CI: cardiac index; eGFR: estimated glomerular filtration rate; mPAP: Mean pulmonary arterial pressure; CPV: central venous pressure; PH: pulmonary hypertension; PVR: pulmonary vascular resistance.*

Several PH hemodynamic parameters (mPAP, CVP, PVR) assessed at 6 weeks showed a significant inverse correlation with renal function (eGFR) (Fig. 3). On the contrary, we did not observe any correlation between CI and renal function.

## Methods

### Animals and experimental design

**Figure 4:**
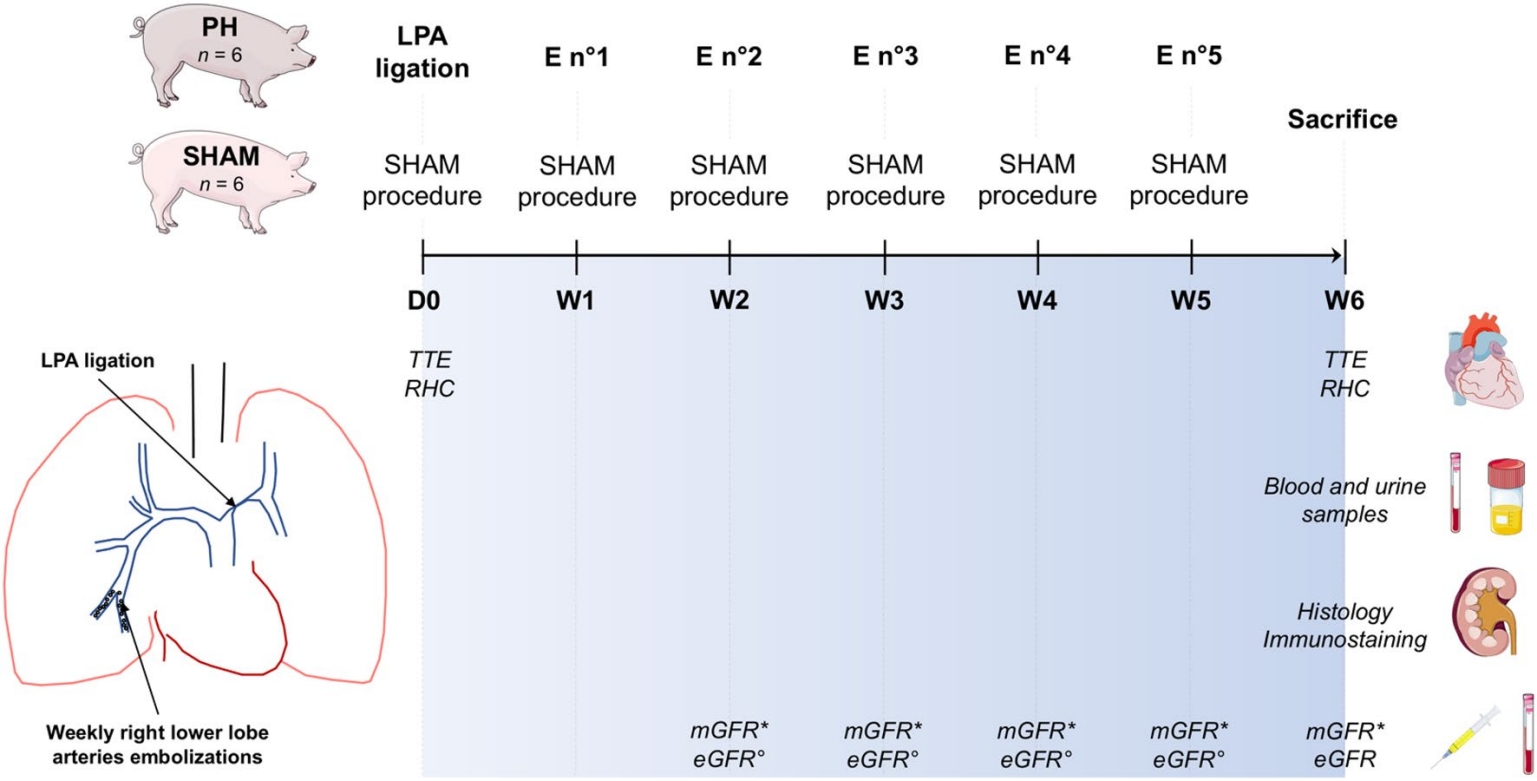
Study protocol of pulmonary hypertension induction. The study protocol consisted of ligation of the left pulmonary artery (LPA) at day 0 (D0) followed by weekly (W) embolization (E) of *n*-butyl-2-cyanoacrylate in the right lower pulmonary artery. * mGFR were carried out weekly on one piglet of group PH from embolization number 2 (W2) until sacrifice. ° eGFR were carried out weekly on one piglet of group PH from embolization number 2 (W2) until sacrifice, and for all piglets at sacrifice. D: day; E: embolization; mGFR: measured glomerular filtration rate; LPA: left pulmonary artery; PH: pulmonary hypertension; RHC: right heart catheterization; TTE: transthoracic echocardiography; W: week.

After one week of acclimatization in the animal facility, twelve 2-month-old Large White female piglets were randomized to a Sham protocol (control group; n = 6) or a 6-week PH protocol induction (PH group; n = 6) (Fig. 4). No inclusion and/or exclusion criteria were established a priori. The study was carried out without blinding. All procedures were performed under sterile conditions. Surgical equipment was sterilized after each intervention in the laboratory. After completion of the study, animals were euthanized using lethal potassium infusion. This study in reported in accordance with the ARRIVE guidelines.

The study complied with “Guidelines for the Care and Use of Laboratory Animals” developed by the National Institutes of Health and with the “Principles of Laboratory Animal Care”, developed by the National Society for Medical Research. The protocol was authorized by the French Ministry of Research after approval by the ethic committee on animal experiments of Paris Saclay University, France (APAFIS#27670-2020101316513,677 v2).

### Induction of pulmonary hypertension and Sham protocol

At day-0 (D0), the first step consisted of a proximal complete ligation of the intrapericardial left pulmonary artery (LPA) after median sternotomy using a non-resorbable suspensory lac (*Surgical Loop 75 cm, 4 mm, B-Braun, Tuttlingen, Germany*). After rigorous hemostasis, a pleural drain was placed and removed after closing the thoracotomy in 3 planes using slow-resorbing braided sutures (*Vicryl)*. The first stage in the control group (Sham procedure) consisted of a median sternotomy on D0 without LAPG. Only the Large White piglets in the PH group had LAPG. The second step started one week later: the right lower lobe arteries were embolized. The embolization procedure was repeated weekly for 5 weeks (for a total of 5 embolizations) to ensure chronic right ventricular pressure overload^10–12^. Iterative embolization of the right pulmonary lobe is necessary to obtain progressive occlusion of the segmental arteries of the right lower lobe while avoiding per-procedural cardiac arrest. Animals in the Sham group only received weekly injection of saline solution for 5 weeks without prior ligation of the LPA. All pigs received intraoperative antibiotic prophylaxis with Augmentin (1 g) renewed postoperatively for 72 h. Postoperative analgesia was performed by administering Nalbuphine (Nubain®, 4 mg/20 kg, twice a day) for 4 days.

### Echocardiography and right heart catheterization

Transthoracic echocardiography was performed at baseline and at the time of sacrifice (*Vivid E9; General Electric Medical System, Milwaukee, WI, USA*). LVEF was calculated by the Teicholz method. Right ventricle systolic function was evaluated with tricuspid annular plane systolic excursion (TAPSE) and systolic (s') velocities of the tricuspid annuli (using tissue Doppler analysis).

Pulmonary hemodynamics data were recorded at baseline, during right heart catheterization (RHC), and at the time of sacrifice. A thermistor-tipped 7F catheter (*Edwards LifeSciences, Irvine, CA, USA*) was placed into the right atrium to determine central venous pressure (CVP) and into the right PA to record mean pulmonary artery pressure (mPAP), pulmonary capillary wedge pressure (Ppcw), and cardiac output (CO). The ventilator was transiently turned off during all measurements. Total pulmonary resistance (TPR) levels were calculated as follow: TPR = (mPAP x 80)/CO. Pulmonary vascular resistance (PVR) were calculated from mPAP, Ppcw and CO (PVR = mPAP − Pcpw/CO). The cardiac index (CI) was obtained by normalizing CO at the body surface area (BSA) by the equation developed by Kelley et al.^13^.

$$BSA \left({cm}^{2}\right)=734 \times {weight}^{0.656}$$

### Blood and urine samplings

At the time of sacrifice, blood was sampled from a peripheral venous access into prechilled vacutainer tubes for analysis. Urine was sampled from the bladder by puncture after laparotomy. Renal function was assessed by measuring plasma urea, serum creatinine (SCr) concentration (enzymatic method), and albuminuria. Myocardium injury was determined by measurement of the plasma concentration of creatine kinase (CK), cardiac troponin I, lactate dehydrogenase (LDH), and aspartate aminotransferase (AST).

After sampling in sterile tubes with lithium heparin (blood samples) and sterile tube (urine samples), the tubes were immediately placed on ice and centrifuged at 2000 rpm, at 4 °C, for 12 min, before the plasma or serum aliquots were frozen at -20 °C until analysis.

Laboratory measurements were performed on an Atellica solution™ analyzer, with reagents from the manufacturer (Siemens Healthcare Diagnostics, Saint Denis, France).

The estimated GFR (eGFR) was determined from weight and SCr by the formula of Gasthuys et al.^14^, validated in young growing piglets:

$$eGFR (ml/min/kg)= \frac{1.879 \times {weight}^{1.092}}{{serum\,creatinine}^{0.6}}$$

### Masson’s trichrome

The kidneys were harvested by laparotomy immediately after the cardiocirculatory arrest in order to minimize the risk of injury induced by hypoperfusion and ischemia. 3-µm sections (manual microtome, *ThermoScientific, MA, USA*) of kidney biopsies fixed in AFA (Alcohol Formalin Acetic acid) and paraffin-embedded were stained in Harris hematoxylin solution for 3 min, then lithium carbonate solution for 10 s and solution of Fuchsin de Ponceau for 3 min. After washing with 1% acetic acid, the sections were immersed in 1% phosphomolybdic acid for 2 × 10 min. Again, they were differentiated in a 1% acetic acid solution and immersed in a 1% green light solution for 10 min. Kidney biopsy sections were blindly examined with a quantitative analysis of tubular lesions by an experienced pathologist (DBu). A semi-quantitative score of the tubular injury lesions of acute tubular necrosis (ATN) was established (from no lesions: 0, to numerous tubular lesions: 3).

### Immunostaining

Ki67 is a marker of cell proliferation^15^. Immunohistochemistry was performed on AFA-fixed and paraffin-embedded kidney biopsies. The biopsies were sectioned into consecutive 3-μm-thick slices on Superfrost Plus glass slides (*Thermo Fisher Scientific, Merelbeke, Belgium*). Before staining, slides were deparaffinized, and antigen retrieval was performed by incubating in sodium citrate buffer (pH 6.0) in a water bath under pressure for 20 min. The sections were blocked with phosphate-buffered saline containing 10% BSA and 0.2% triton and then incubated overnight with an anti-Ki-67 monoclonal antibody (*SolA15*), coupled with the eFluor™ 660 fluorophore (1/200, *50–5698-82, Ebioscience, ThermoFischer Scientific, MA, USA*). Negative controls were performed by omitting the primary antibody. After 3 phosphate-buffered saline rinses, the sections were incubated for 3 min with DAPI to stain all cell nuclei (1/4000, *62248 ThermoFisher Scientific, MA, USA*). Slides were mounted with coverslips (*Menzel-Gläser*) and mounting medium (*Immu-mount, 9990402 Epredia Western Michigan University College of Engineering and Applied Sciences, USA*).pH2AX (*phospho-H2AX or gamma-H2AX*) is a marker of DNA damage. Immunofluorescence staining was performed with the rabbit monoclonal antibody against pH2AX-S139 (1/200, *APO 687, AB clonal, MA, USA*) overnight. After 3 phosphate-buffered saline rinses, the sections were incubated for 30 min with the secondary antibody (*Invitrogen Alexafluor647 goat anti-rabbit ref: A21245*). We used DAPI to stain all cell nuclei for 3 min. Slides were mounted with coverslips and mounting medium.

Automatic quantification of Ki67 and pH2AX immunostainings was performed with QuPath^16^.

### Transdermal measurement of glomerular filtration rate (mGFR)

A miniature fluorescence detector was attached to the skin on the back of piglets during its general anesthesia (*MediBeacon®, MO, USA*)^17^. An exogenous fluorescent tracer with renal elimination (relmapirazin) was injected as a bolus by peripheral venous access and is distributed in the vascular system, then in the tissue system and is eliminated by the kidney. The transdermal detector records a peak concentration of the tracer followed by a slow decrease allowing estimation of the mGFR and the elimination half-life of the tracer. These evaluations were carried out weekly on a pig of group PH from embolization number 2 (W2) until sacrifice.

### Statistical analyzes

Statistical analyzes were performed using GraphPad Prism 9.0.0 (*GraphPad Software, San Diego, California, USA*). Descriptive statistics include the mean ± standard deviation (SD) or the median with [interquartile ranges] (IQR) for continuous variables. Quantitative variables were compared using an unpaired t-test or with a Mann–Whitney test for nonparametric analysis. Correlations between renal function and hemodynamic parameters of PH were performed using linear regression models. A *p-*value < 0.05 was considered significant.

### Ethics approval and consent to participate

The study complied with “Guidelines for the Care and Use of Laboratory Animals” developed by the National Institutes of Health and with the “Principles of Laboratory Animal Care”, developed by the National Society for Medical Research. The protocol was authorized by the French Ministry of Research after approval by the ethic committee on animal experiments of Paris Saclay University, France (APAFIS#27670-2020101316513677 v2).

## Discussion

We report here the first porcine model of CRS secondary to right ventricular pressure overload due to PH. RHC confirmed PH as an increase in mPAP greater than 20 mmHg and PVR higher than 3 WU, and we observed an increase in CVP without a decrease in CI. PH was responsible for right ventricular dysfunction objectified by systolic dysfunction (decrease in TAPSE) with myocardial distress marked by an increase in cardiac troponin I. Taken together, these data demonstrated that the modelization of PH in piglets resulted in significant right ventricular pressure overload associated with right ventricular systolic dysfunction. Renal dysfunction secondary to right cardiac dysfunction was objectified in the PH group and characterized by tubular histological lesions and an increase in the expression of the DNA damage marker pH2AX in renal tubular cells. The increase in DNA damage in the PH group did not seem to pertain to an increase in DNA synthesis as we found no difference in the Ki67 expression between the Sham and the PH groups. Biologically, the PH piglets exhibited significantly increased albuminuria, reduced urea excretion fraction, and a decrease in GFR in the one piglet which ad a longitudinal assessment of mGFR during the PH induction protocol.

### Renal dysfunction due to PH

The usual markers of renal dysfunction (SCr, plasma urea, or eGFR from the SCr) do not make it possible to demonstrate renal insufficiency and seem to lack the sensitivity to detect an alteration of renal function in our model. Several reasons can explain these results. First, our model was developed on growing piglets whose weight and muscle gain were significant during the follow-up (8.4 ± 3.1 kg in the PH group and 9.5 ± 3.1 kg in the Sham group), which could have biased the SCr and eGFR interpretation. In addition, as in humans, piglets undergo postnatal renal maturation during growth marked by an increase in GFR during the first weeks of life^14,18^. Finally, an AKI involves renal damage (tubular injuries or albuminuria) but not necessarily dysfunction (eGFR decrease) because the kidney has a large reserve of glomerular function^19^. It would be interesting to assess the value of biomarkers of tubular damage (NGAL, KIM-1, TIMP2/IFGBP7) in this particular setting of PH.

Estimating GFR from SCr (such as Gasthuys et al. formula) is clinically relevant but has some limitations. These formulas lack sensitivity and cannot be used when body height may not accurately reflect muscle mass or if GFR is rapidly changing (AKI). Thus, as observed in our model, the measurement of GFR by an exogenous tracer is the most sensitive and precise method for determining renal function^20^. Measurement of transdermal GFR with an exogenous fluorescent tracer is an innovative method validated in small animals that allows rapid and accurate measurement of GFR without blood or urine sampling^21^. We report in this study one of the first iterative evaluation of this device in piglets^17^.

The increase in albuminuria observed in our study appears to confirm the results of two human studies that show a moderate increase in albuminuria following right ventricular dysfunction associated with PH^22,23^. Although the mechanisms are not fully understood, altered glomerular hemodynamics and permeability (due to venous congestion) and the systemic inflammation in PH could contribute to increased albuminuria, but low levels of albuminuria can also be caused by defective epithelial reabsorption of filtered proteins in purely tubular injury^24^.

### Cardiac dysfunction and venous congestion impact

Elevated cardiac troponin is observed in right ventricular overload, as in PH. In humans, elevated plasma troponin I is associated with more severe disease and worse outcomes in patients with PH^25^.

While our team has solid experience with this piglet model, we observed heterogeneity of cardiac injury. For example, we noted significant increases in mPAP and PVR (32 ± 10 mmHg, 9.3 ± 4.7 mmHg, respectively) in the PH group. We observed some heterogeneity in PH severity. These differences may have partly blunted the effect of our model on renal function.

We reported a linear correlation between hemodynamic parameters of PH (mPAP, CVP, and PVR) and renal dysfunction (eGFR). These observations concur with the results observed in humans: venous congestion (increase in CVP) in patients with PH is strongly correlated with GFR^7^. Renal congestion resulting from PH due to chronic pressure overload is characterized by a decrease in the transcapillary glomerular pressure gradient, a reduction in renal perfusion pressure (drop in the fraction of urea excretion in our study), and GFR reduction^26,27^. We do not observe neither clinical features of right heart failure (RHF) nor significant decrease in CI. No correlation was found between CI and renal dysfunction. Our PH model rather induced compensated remodeling with moderate systolic dysfunction than right ventricular failure. However, this model has been demonstrated to induce significant impairment in right ventricular-pulmonary arterial coupling, consistent with right ventricular dysfunction^28^. Thus, venous congestion appears to be the most critical hemodynamic factor in renal dysfunction during PH in our model. Our model allows to evaluate the effect of PH on the kidney, particularly the probable deleterious effect of venous congestion, without having the effect related to low cardiac output.

### New perspectives

A comparison with a second model of left cardiac dysfunction could be interesting. The current classification of CRS is based on the timing of organ dysfunction (acute or chronic), while the pathophysiology of types 1 and 2 are similar. In contrast, to our knowledge, no study has compared a model of LHF to a model of RHF, which has different pathophysiological characteristics^29^. Thus, we could evaluate the differential effect of therapies based on primary cardiac involvement (RHF or LHF) or on renal congestion. Future research is needed to evaluate the efficacy of vasoactive drugs in preventing renal dysfunction secondary to right ventricular pressure overload associated with right ventricular systolic dysfunction by exposing the Large White piglets to nitrates, loop diuretic, sodium/glucose cotransporter inhibitor (iSGLT2)^30^, or RAAS inhibitor, alone or in combination with vasoactive drugs to maintain the central blood pressure during the PH induction protocol.

## Conclusion

We report here the first porcine model of CRS secondary to right ventricular pressure overload due to PH.

## Data availability

The dataset used and analyzed for the current study is available from the corresponding author on reasonable request.

## Supplementary Information

Supplementary Information.

## Abbreviations

AFA: Alcohol formalin acetic acid
AST: Aspartate aminotransferase
CI: Cardiac index
CK: Creatine kinase
CO: Cardiac output
CRS: Cardiorenal syndrome
CVP: Central venous pressure
D0: Day 0
E: Embolization
eGFR: Estimated glomerular filtration rate
GFR: Glomerular filtration rate
IQR: Interquartile ranges
LDH: Lactate dehydrogenase
LHF: Left heart failure
LPA: Left pulmonary artery
LVEF: Left ventricular ejection fraction
mGFR: Measured of glomerular filtration rate
mPAP: Mean pulmonary artery pressure
PH: Pulmonary hypertension
PVR: Pulmonary vascular resistance
RAAS: Renin–angiotensin–aldosterone system
RAP: Right atrial pressure
RBF: Renal blood flow
RHC: Right heart catheterization
RHF: Right heart failure
SCr: Serum creatinine
SD: Standard deviation
TAPSE: Tricuspid annular plane systolic excursion
TTE: Transthoracic echocardiography
TPR: Total pulmonary resistance
W: Week
