# Supplementary material for: Pulmonary hypertension without heart failure causes cardiorenal syndrome in a porcine model
Source: Sci Rep. 2023 Jun 5;13:9130. doi: 10.1038/s41598-023-36124-1 (PMC10241877; doi:10.1038/s41598-023-36124-1)
Supplement: Supplementary file 1 — Supplementary Information. [file 41598_2023_36124_MOESM1_ESM.docx]

**SUPPLEMENTARY MATERIAL**

**Figure S1. Representative immunostainings for pH2AX and Ki67.**

**Table S1. Hemodynamic and echocardiographic parameters at baseline evaluation.**

*CI: cardiac index; LVEF: left ventricular ejection fraction; mPAP: Mean pulmonary arterial pressure; PVR: pulmonary vascular resistance; TAPSE: tricuspid annular plane systolic excursion; TPR: total pulmonary resistance*

|  | **PH (*n* = 6)** | **SHAM (*n* = 6)** | **p-value** |
| --- | --- | --- | --- |
| ***Hemodynamic parameters*** | | | |
| Heart rate (bpm) | 106 [101-121] | 112 [103-127] | 0.73 |
| Mean pulmonary artery pressure (mPAP) (mmHg) | 14.7 ± 2.1 | 13.3 ± 4.2 | 0.50 |
| Total pulmonary resistance (TPR) (WU) | 4.7 ± 1.1 | 3.6 ± 0.4 | 0.10 |
| Pulmonary vascular resistance (PVR) (WU) | 3.6 ± 3.1 | 2.3 ± 1 | 0.21 |
| Cardiac index (CI) (L/min/m^2^) | 3.3 ± 0.9 | 3.6 ± 0.9 | 0.59 |
| ***Echocardiography parameters*** | | | |
| LVEF (%) | 70.3 ± 3.1 | 68.8 ± 4.2 | 0.49 |
| Tricuspid S’ peak systolic velocity (cm/s) | 9 [8-12] | 8 [8-10] | 0.53 |
| TAPSE (cm) | 15 [15-16] | 14 [13-14] | 0.05 |
